# Microscale Thermophoresis and Molecular Modelling to Explore the Chelating Drug Transportation in the Milk to Infant

**DOI:** 10.3390/molecules27144604

**Published:** 2022-07-19

**Authors:** Mufarreh Asmari, Muhammad Waqas, Adel Ehab Ibrahim, Sobia Ahsan Halim, Ajmal Khan, Ahmed Al-Harrasi, Hermann Wätzig, Sami El Deeb

**Affiliations:** 1 College of Pharmacy, King Khalid University, Abha 62529, Saudi Arabia; masmri@kku.edu.sa; 2 Natural and Medical Sciences Research Center, University of Nizwa, P.O. Box 33, Birkat Al Mauz, Nizwa 616, Oman; mwaqas@unizwa.edu.om (M.W.); adel.ehab@pharm.psu.edu.eg (A.E.I.); sobia_halim@unizwa.edu.om (S.A.H.); ajmalkhan@unizwa.edu.om (A.K.); aharrasi@unizwa.edu.om (A.A.-H.); 3 Analytical Chemistry Department, Faculty of Pharmacy, Port-Said University, Port Fouad 42526, Egypt; 4 Institute of Medicinal and Pharmaceutical Chemistry, Technische Universität Braunschweig, 38106 Braunschweig, Germany; h-waetzig@tu-bs.de

**Keywords:** microscale thermophoresis, lactoferrin, deferiprone, dissociation constant, molecular modeling

## Abstract

The microscale thermophoresis (MST) technique was utilized to investigate lactoferrin–drug interaction with the iron chelator, deferiprone, using label-free system. MST depends on the intrinsic fluorescence of one interacting partner. The results indicated a significant interaction between lactoferrin and deferiprone. The estimated binding constant for the lactoferrin–deferiprone interaction was 8.9 × 10^−6^ ± 1.6, SD, which is to be reported for the first time. Such significant binding between lactoferrin and deferiprone may indicate the potentiation of the drug secretion into a lactating mother’s milk. The technique showed a fast and simple approach to study protein–drug interaction while avoiding complicated labeling procedures. Moreover, the binding behavior of deferiprone within the binding sites of lactoferrin was investigated through molecular docking which reflected that deferiprone mediates strong hydrogen bonding with ARG121 and ASP297 in pocket 1 and forms H-bond and ionic interaction with ASN640 and ASP395, respectively, in pocket 2 of lactoferrin. Meanwhile, iron ions provide ionic interaction with deferiprone in both of the pockets. The molecular dynamic simulation further confirmed that the binding of deferiprone with lactoferrin brings conformational changes in lactoferrin that is more energetically stable. It also confirmed that deferiprone causes positive correlation motion in the interacting residues of both pockets, with strong negative correlation motion in the loop regions, and thus changes the dynamics of lactoferrin. The MM-GBSA based binding free energy calculation revealed that deferiprone exhibits ∆G TOTAL of −63,163 kcal/mol in pocket 1 and −63,073 kcal/mol in pocket 2 with complex receptor–ligand difference in pocket 1 and pocket 2 of −117.38 kcal/mol and −111.54 kcal/mol, respectively, which in turn suggests that deferiprone binds more strongly in the pocket 1. The free energy landscape of the lactoferrin–deferiprone complex also showed that this complex remains in a high energy state that confirms the strong binding of deferiprone with the lactoferrin. The current research concluded that iron-chelating drugs (deferiprone) can be transported from the mother to the infant in the milk because of the strong attachment with the lactoferrin active pockets.

## 1. Introduction

Lactoferrin (Lf) is known as a multifunctional protein that possesses antimicrobial, antitumor, anti-inflammatory, protease inhibition and iron chelation effects [1]. Lactoferrin is present in mammalian milk as a nutrient. Several drugs have been paid more attention to during the current SARS-CoV-2 pandemic (COVID-19) in order to be repurposed as potential treatment protocols [2,3]. Lf, as a natural supplement, has shown to be helpful in the treatment of COVID-19 infected patients [1,4,5]. Furthermore, the molecular mechanisms of Lf as anti-SARS-CoV-2 have been explored [6]. Lf is also present naturally in all biological fluids; thus, it is considered a good targeted protein for interaction studies of drug pharmacokinetics, drug efficacy and drug design [7,8]. Deferiprone (CP20), 1,2-dimethyl-3-hydroxypyrid-4-one, is the first orally active iron-chelator that was approved for the treatment of iron overload syndrome correlates to β-thalassemia [9]. The three-dimensional structures of human’s [10,11] and rabbit’s [12] Lf protein were first resolved by X-ray crystallography. The Lf polypeptide has an N-terminal half forming one lobe (N-lobe), and a C-terminal half the other (C-lobe), which makes the protein bilobal. The combined metal and anion sites are located in a deep gap between two domains in each lobe. The two metal-binding locations in Lf protein are substantially similar, where the Fe^3+^ and CO_3_^−^ are bonded.

CP20 is capable for sequestering non-transferrin bound iron within the targeted sites since iron is vital for several biosynthetic and transport pathways due to its characteristic redox chemistry. CP20 is a bidentate chelator which binds with Fe^3+^ under the physiological conditions to form a Fe-(CP20)_3_ complex in a 1:3 binding ratio. CP20 is still promising as iron chelator in several clinical situations, other than iron overload syndrome. Those situations include cancer, fungal and viral infections, and renal insufficiency [13]. Moreover, CP20 is presently in phase II clinical trials for treatment of Parkinson’s syndrome [14] and reported to be beneficial in the treatment of Friedreich’s ataxia [15]. CP20 is able to penetrate into the biomolecules and induce effects, which might be either desirable or undesirable. This interaction can alter the metabolic pathways of several metal ions, especially those metal ions which are present in levels that compete with Fe^3+^ for its metal-binding sites [16,17]. For instance, CP20 has been reported to interact with Lf [16], hemoglobin [18], and human serum albumin [19]. 

**Figure 1:**
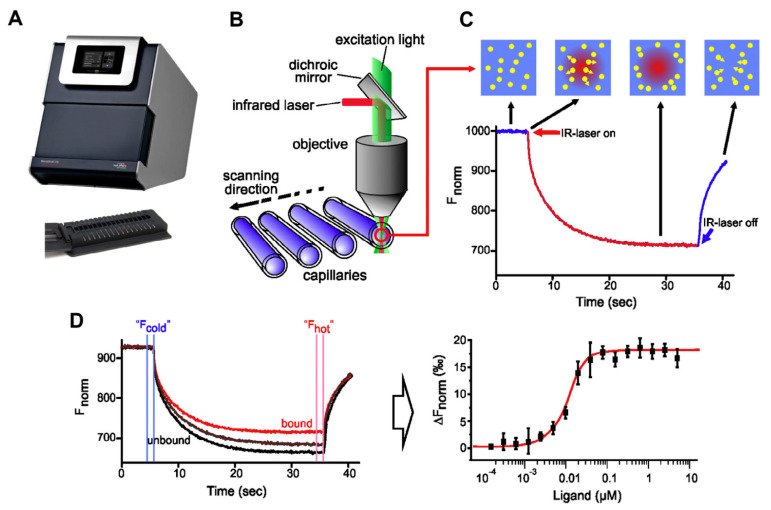
Principle of MST instrument (**A**–**D**), reprinted with permission from reference [23].

Label-free microscale thermophoresis (MST) is a relatively new promising technique that is used to measure molecular interactions through an induced temperature gradient in microscale size. In MST, thin capillaries containing molecules of microscopic size (50 µM) are subjected to an IR laser. This later induces a temperature gradient where those molecules escape from heat (thermophoresis phenomenon). This thermophoretic movement of molecules is affected by some molecular properties such as molecular size, conformation and/or charge. MST requires that one of the interacting molecules have a fluorescent character to be measured during the thermophoretic movement. When the speed of migration of free the target molecule is changed upon addition of ligands, this indicates bio-molecular interaction which can be measured by MST with no restriction to the buffering system or immobilization procedures. Eventually, the binding of ligand to the target molecules will at least change one of its molecular parameters [20,21,22]. The principle of MST is schematically presented in Figure 1. 

In this study, since Lf is the most widely found metalloprotein in mammalian breast milk, the investigation of CP20 interaction with Lf would be valuable to assess the potential drug secretion in breast milk as a function of the bound fraction to Lf, as well as iron chelation synergism. Herein, the interaction study of CP20 with Lf was conducted using MST, while molecular modelling was employed to investigate the binding parameters for the first time.

## 2. Results and Discussion

### 2.1. Microscale Thermophoresis

**Figure 2:**
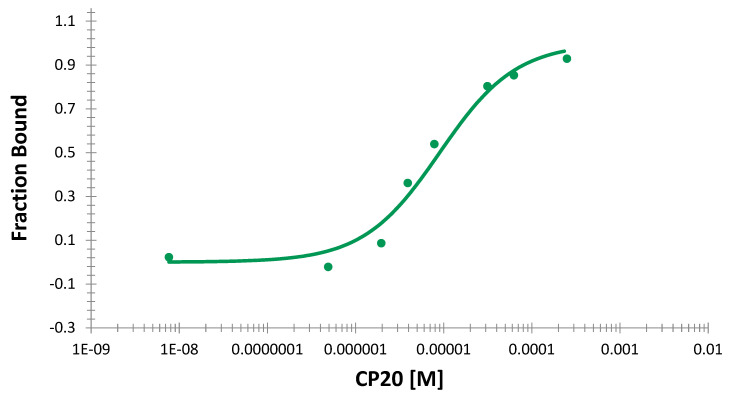
The binding curve of the CP20−Lf interaction using the MST method (Kd = 8.9 × 10^−6^ Molar ± 1.6, SD).

The MST method is considered noninvasive and capable of quantifying the binding parameters under similar native conditions. During MST scanning, Lf exhibited extremely strong fluorescence signals in comparison to those of human serum albumin (HSA), which might be attributable to the number of tryptophan residues. While Lf contains nine tryptophans, HSA contains only one residue. The proper concentration for Lf to obtain sufficient fluorescence signals was selected to be 120 nM, which was in the role of the fluorescent partner. Conversely, CP20 was prepared at different concentrations in the range of 7 nM to 250 μM. The MST data were fit with the dissociation constant (*K_d_*) model and the estimated Kd value was in low micromolar concentration with 8.9 × 10^−6^ Molar ± 1.6, SD. The binding isotherm is shown in Figure 2.

### 2.2. In Silico Analysis

#### 2.2.1. Lactoferrin Oxalate Ion and Deferiprone Complexes

**Figure 3:**
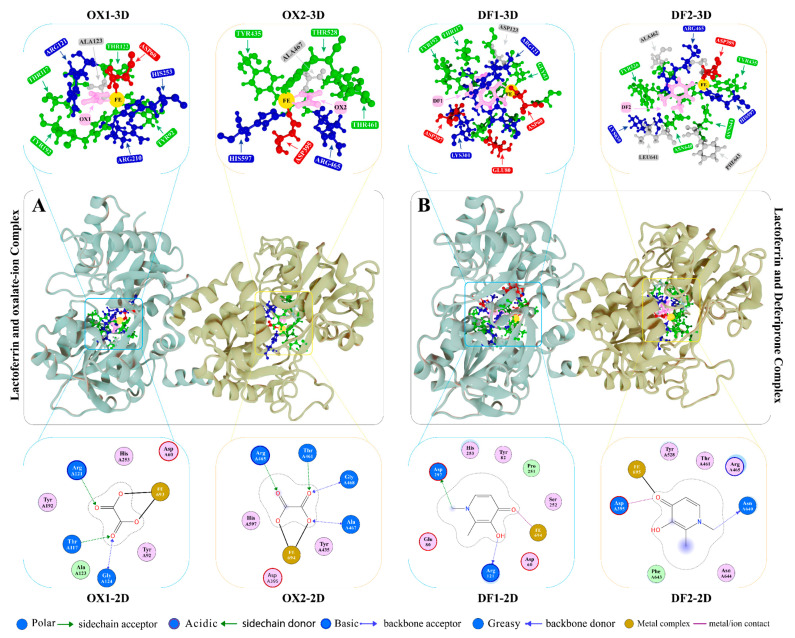
The 3D structure of lactoferrin is presented (cartoon shape) in complex with (**A**) co-crystallized oxalate ion (PDB ID: 1BKA), and (**B**) deferiprone.

**Figure 4:**
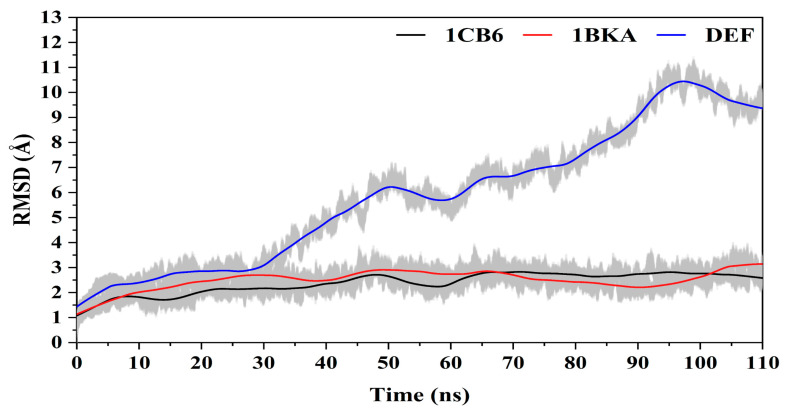
The graphical presentation of root mean square deviation in 1CB6, 1BKA, and DEF.

In the crystal structures of Lf, oxalate ion is attached in two pockets of the protein with Fe ion with +3 charges (Figure 3). The oxalate ion interacts in pocket 1 (OX1) with side chains of THR117, GLY124, and ARG121 by a hydrogen bond with a 2.7 Å distance, while the acidic residue, ASP60, forms ionic interaction with the Fe ion at 1.9 Å distance and −3.8 kcal/mol energy. The pocket 2 (OX2) of Lf comprises ALA467, THR461, GLY468, ARG465, and ALA467 that form H-bonds with the oxalate ion at 2.8–2.9 Å and the ASP395 form an ionic interaction with Fe^3+^ ion with interaction energy of −4.1 kcal/mol. The docked conformation of CP20 in pockets 1 (DF1) and 2 (DF2) showed stable interactions with the active site residues and the Fe ion with a docking score of −4.85 kcal/mol and −4.54 kcal/mol, respectively (Figure 4). The RMSD of deferiprone in pockets 1 and 2 was 0.362 Å and 0.767 Å, respectively, with oxalate ion. The re-docking of the co-crystallized ligand (oxalate ion) showed a root mean square deviation (RMSD) of 0.265 Å and a docking score (DS) of −4.25 kcal/mol. The RMSD is in an acceptable range, thus validating the docking protocols. The DF1 pocket has H-bond donors (ARG121 and ASP297) that mediate H-bonding with deferiprone, while Fe^3+^ ion form ionic interactions with THR122 and ASP60 at 2 Å and a 1.9 Å, respectively. Correspondingly, in pocket 2 (DF2), CP20 interacts with the ASN640 through H-bond (3 Å) and through ionic interaction with ASP395 (2.1 Å). Fe^3+^ ion in DF2 forms an ionic interaction with ASP395 and HIS597. Fe^3+^ ion showed a strong interaction (−18.2 kcal/mol) with the ASP395 residue and the deferiprone. The docking analysis concluded that DEF showed a stable binding with Lf’s active pocket. The detailed interactions of both ligands are tabulated in Supplementary Tables S1 and S2.

#### 2.2.2. Stability of the Molecular Dynamics Simulation

The structural characteristics of Lf from the molecular dynamic (MD) simulation is vital to understand the inhibitory mechanism. The free form (1CB6) of Lf (apo-Lf) was simulated with the reference inhibited state (1BKA, Lf–oxalate ion complex) and compared with the Lf docked CP20 complex. The structural stability was compared by the RMSD of the apo-Lf and inhibited states of the Lf from the 110 ns trajectories (Figure 4). The apo-Lf (1CB6) showed a stable RMSD between the 1.5 Å to 2 Å with small periodic jumps concluding that the apo-Lf was converged. The reference inhibited complex (1BKA) showed a small variation in the RMSD pattern (average 2 Å RMSD) in the simulation. The small molecule attached complex converged during the simulation. Comparing the Lf- CP20 complex (DEF) with 1CB6 and 1BKA, during the first 30 ns, the RMSD pattern of DEF was same (2 Å) like 1BKA; however, movement was increased after 30 ns and RMSD was raised to 6 Å till 50 ns. In addition, a steep increase in RMSD (10 Å) was observed from 50 ns to 95 ns. From 95 ns to 110 ns, DEF converged its RMSD by normalizing it to decrease. Due to the binding of DEF in the active pockets of the Lf, the DF1 pocket moves in opening conformation which increased the protein RMSD. The RMSD graph reported no sudden deviation, and this confirmed the significance of the simulation results.

Pocket 1 and pocket 2 are shown in light blue and light yellow ribbons, respectively. The interactions (3D format) in both pockets are represented in the cartoon structure, while the 2D interaction diagrams are represented below. 

#### 2.2.3. Binding Free Energy Calculations

The MM-GBSA method was used to calculate the binding free energy between the Lf with oxalate ion and CP20 with individual components energies required in the binding free energy calculations (Table 1). Both pockets were analyzed separately to calculate the binding energy of each ligand in the active site, and the energy components which contribute to the binding free energy were calculated. The free energy of the protein–ligand complex was estimated from 1000 frames of the 110 ns trajectory of each system. Energy calculation reveals the difference in the energies of the bound oxalate ion and docked deferiprone. The OX1 pocket oxalate ion exhibits a total binding free energy of −129.32 kcal/mol considered to be the highest, while the oxalate ion attached in the OX2 pocket shows binding energy of −126.78 kcal/mol, which suggests that the oxalate ion attach in OX1 pocket with more strength than the OX2 pocket. However, the DEF complex exhibited binding energy of −117.38 kcal/mol in the DF1 pocket, while the DF2 pocket DEF calculated binding free energy was −111.54 kcal/mol, respectively. DEF complex with Lf in DF1 and DF2 suggests that CP20 attaches more strongly in DF1 than DF2 pockets. The ligands (oxalate ion and deferiprone) bound complexes are considered energetically favorable from the MM-GBSA analysis. The binding energy calculations concluded that the DEF binding in the LF shows a firm interaction with the active site residues and Fe^3+^ ion. 

#### 2.2.4. Protein–Ligand Interaction Analysis

The native contacts were analyzed in both complexes (Lf with oxalate ion and CP20) to investigate the stability of the Fe binding with the ligand and active site residues. The ionic interaction of oxalate ion with Fe was reported 100% fraction in both pockets (OX1 and OX2) during simulation (Supplementary Table S2). Moreover, CP20 followed the same pattern in both pockets, DF1 and DF2, where Fe ion formed ionic interaction with 100% fraction rate (Supplementary Table S3). The highest interaction fraction shown by the active site residues with ligand in OX1 pocket are the residue PHE190 (99.90% fraction with an average distance of 2.68 Å) and LEU119 (two interactions with 62% and 53% fraction rate). In the OX2 pocket, ASP464 showed highest interaction fraction (37.40%) with the oxalate ion and THR90 (fraction rate of 2.02%) also displayed good binding distance (2.7 Å). Other interactions with fractions < 1% in the OX1 were formed by THR90, CYS115, ARG121, and PHE190. Whereas in the OX2, VAL463 showed the second highest binding fraction (30.20%) with an average bond distance of 2.8 Å while TYR526 (13.20%), SER458 (12.50%), and ALA462 and PRO595 showed 11% fraction rate. The residues VAL463 and GLU433 of OX2 have 8% fraction rate, while TYR525 formed two interactions with the oxalate ion with 5–6% fraction rate. The interactions of oxalate ions in both pockets revealed that OX1 has high fraction rates of bonds than OX2 during the simulation. 

In the case of CP20, the highest fraction in the DF1 was shown by VAL81 with a 96.90% fraction rate with an average distance of 2.7 Å, similarly, VAL81 in the DF1 demonstrated the second-highest lifespan of the bond with deferiprone, while TYR93 showed 4.59% bond life with the CP20. Whereas GLY83 reflected below 1% fraction rate in the DF1. While in the DF2, LEU642 (54.50%) showed the highest interaction fraction with deferiprone with an average bond distance of 2.8 Å. Afterwards, ASP645 is the DF2 has a 35.30% bond fraction rate with an average bond distance of 2.6 Å. While TYR528 showed the third highest bond fraction in the DF2. However, SP464 and ARG643 also showed interactions but <1% lifespan. These contacts suggest that the protein–ligand and Fe ion interactions are stable and have an effect on the protein structural dynamics, which concluded that the DEF interact strongly with the Lf active pockets.

#### 2.2.5. Per Residue Energy Decomposition (PRED)

The impact of each residue in the active site of Lf on the binding of oxalate ion and CP20 was further explored by per residue energy decomposition (PRED) to calculate the contribution of the binding energy of each residue in the total binding energy (Supplementary Table S4). The results showed that the Fe ion has the highest contribution to the total binding energy when attached to the oxalate ion (Δ*G* total −58.057 kcal/mol) in the OX1 pocket, whereas three residues including ARG119, THR120, and TYR190 in the OX1 also exhibited the highest binding energy. In the OX2, the Fe ion also contributed the highest energy of (−44.056 kcal/mol), subsequently, LEU433 and THR526 also contributed high in total binding energy, followed by THR463, ALA459 and ALA595. 

Like OX1 and OX2, the contribution of Fe to the binding energy was highest in the DF1 (−30.574 kcal/mol) for CP20 followed by Asp61, GLU81, TYR93, TYR83, HIS254 and VAL82. Similarly, Fe has the highest binding contribution (−28.494 kcal/mol) to the total binding energy of the Lf–CP20 complex in DF2, whereas the top contributing residues of the DF2 are THR531 and LEU643, followed by GLY398 and ALA464. Hence, the contribution of these residues confirms the selectivity of the residues which is crucial for the attachment to the Fe ion and the ligands (oxalate ion and deferiprone). In supporting the DEF interaction with the important residues where the reference oxalate ion interacts, the strong bonding of the DEF is confirmed. 

#### 2.2.6. Residual Fluctuation and Compactness of the Protein

**Figure 5:**
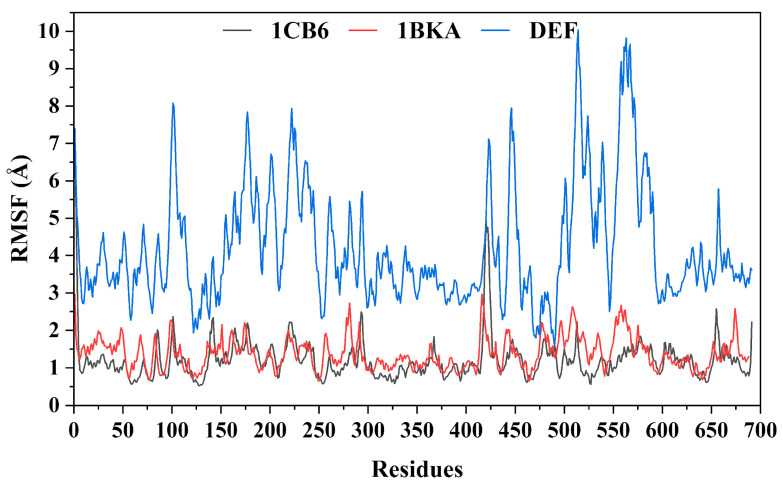
RMSF analysis of Lf in apo form (1 CB6), in oxalate ion bound (1BKA), and in deferiprone bound (DEF) complexes.

The residual flexibility of Lf was estimated through root mean square variations (RMSF). The apo-Lf (1CB6) and the reference bound Lf (1BKA) showed similar pattern of flexibility with a RMSF of 1.2 Å and 1.3 Å, respectively. However, CP20 complex (DEF) displayed high residual flexibility with an average RMSF of 4.2 Å. The increase in flexibility was observed in the active pocket of CP20 due to the movement of some loop regions. The differential dynamics in the flexibility of 1CB6 and DEF is due to the CP20 attachment in both active pockets. Overall, the attachment of oxalate ion and CP20 with Lf increases the residual fluctuation in the active pocket’s regions in the Lf structure (Figure 5). The RMSF analysis determined that the DEF form a strong binding interaction with the Lf which alter the active the protein structural dynamics. 

**Figure 6:**
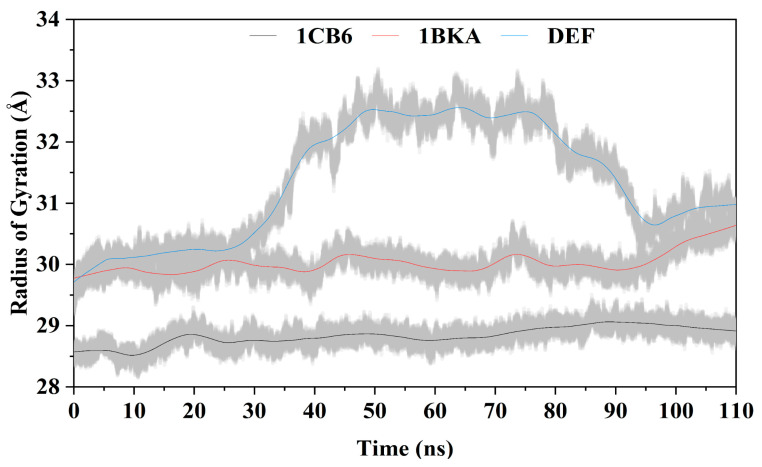
Radius of gyration of the 1CB6, 1BKA, and CP20 complex (DEF).

The Lf atoms mass-weighted RMS distance from the common center was computed by radius of gyration (Rg) which indicates the protein dimensions. The average Rg of 1CB6 was 28.8 Å, with no periodic jumps (Figure 6). The attachment of oxalate ion (1BKA) affects the compactness of Lf by gaining periodic jumps in the overall pattern of Rg with an average value of 30.02 Å. The CP20 complex showed a smooth Rg of ~30 Å up to 30 ns. After 30 ns, a steep increase was observed from 30–33 Å to 65 ns, while after 65 ns, a continuous decrease in Rg was observed (30.5 Å) until 95 ns. The average Rg of CP20 complex was 31.35 Å. The increase in Rg in 1 BKA and CP20 complex reflect that binding of oxalate ion and deferiprone with the active site residues of Lf mediates conformational changes and disturb the initial structure of the protein. The active pockets of the protein were transformed due to the strong bond formation of the DEF with the Lf active pocket residues and Fe^3+^ ions.

#### 2.2.7. Protein Trajectories Motion Clustering

**Figure 7:**
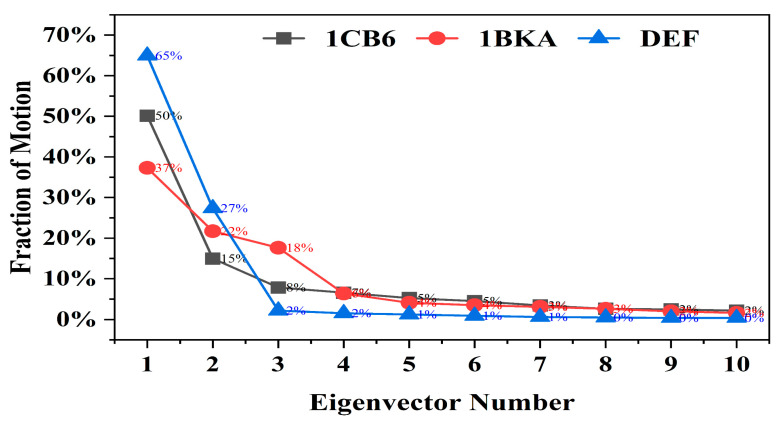
Ten eigenvectors isolated from PCA analysis obtained from covariance matrix showing the fraction of motion in each eigenvector against the corresponding eigenvector constructed from the MD trajectory.

The impact of the binding of oxalate ion and CP20 on the dynamics of Lf was also observed (Figure 7). To understand the structural changes and their amplitude caused by the oxalate ion and CP20, Principal component analysis (PCA) was used and compared with the apo-Lf (1CB6). The first three eigenvectors from the PCA represent the dominant motions, while the other seven eigenvectors show localized motions in the Lf structure. The CP20 complex contributes 65% of the variance in the first eigenvector, while 1CB6 and 1BKA showed 50% and 37% total motion, respectively, which confirms that the small compounds bound complexes adapted different behavior of motion in the protein structure, which reflect the stable binding of small inhibitors in the active sites of 1BKA and CP20 complex. 

**Figure 8:**
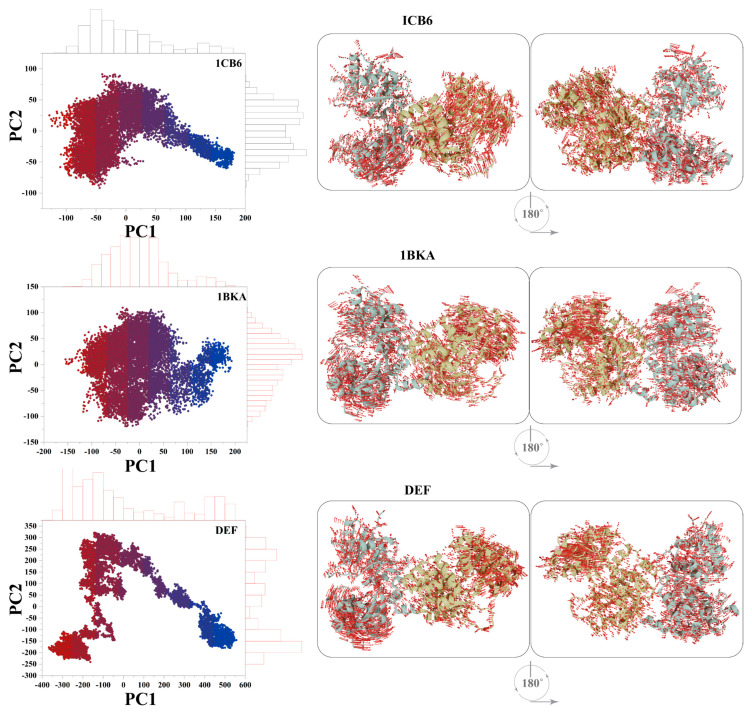
Principal components analysis of 1CB6, 1BKA and CP20 complex (DEF). The motion images are presented in cartoon models. The direction of arrows shows the direction of motion, and length of the arrows shows the magnitude of the motion. Pocket 1 and pocket 2 are shown in sky blue and light yellow color, respectively, in the cartoon model.

Moreover, to obtain the conceivable attributed motions, the first two eigenvectors (PC1 and PC2) were plotted against each other (Figure 8). During the simulation, the flipping over of conformations is shown by blue to red color. Each frame of the simulation in the plot is indicated by a dot start from blue and end on the red color. Each system attains two conformational states (from blue to red). These colors in those two states can easily be separated; where the blue color indicates the unstable conformational state, while the red color shows the stable confirmation. The small molecules attached complexes (1BKA and DEF) showed different transitional periodic jumps. The motions caused by the small molecule attachment in the Lf domains are presented in cartoon shape and show the motion direction and amplitude by the direction and size of the arrow. In the 1CBA, pocket 1 showed opposite direction motion therefore opening the pocket while pocket 2 shows inward motion. These motions in both the pockets were disturbed and changed by the attachment of oxalate ion and deferiprone. In 1BKA and CP20 complex, the directions of the motion were rotated to stabilize the conformation which confirms the strong interaction of the oxalate ion and CP20 in Lf active pockets. 

#### 2.2.8. Protein Correlation Motion

**Figure 9:**
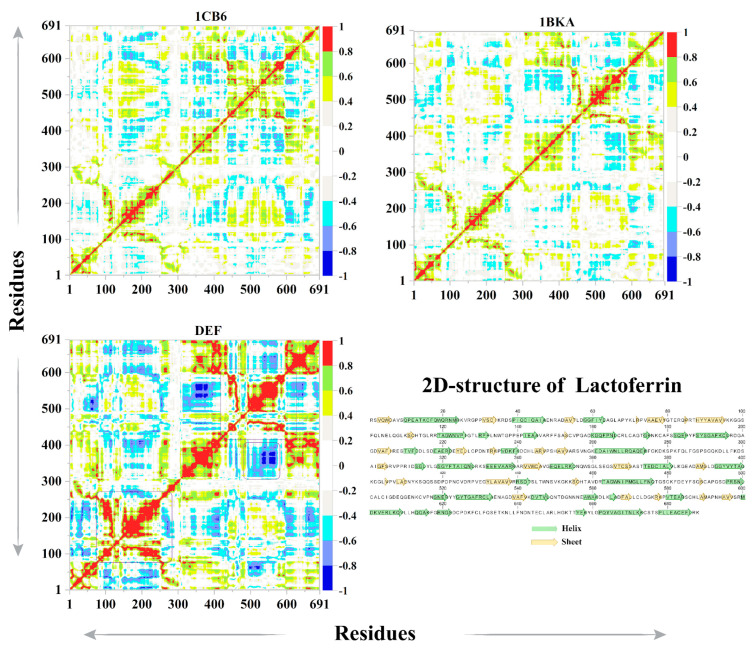
Dynamic cross correlation plot of apo-Lf (1CB6), oxalate-bound Lf (1BKA), and CP20–Lf complex (DEF). In the 2D structure of Lf, helixes, and sheets, are presented in green, and yellow arrows, respectively, and loops are shown as single letter amino acid codes (in black color).

The dynamic cross-correlation matrix (DCCM) was constructed and analyzed for the functional displacement of the protein interacting atoms investigated with simulation time. During the 110 ns simulation time, the CP20 complex showed more positive correlation motion than 1CB6 and 1BKA (Figure 9). The residues ranging from 100 to 200, which consist of the pocket 1 interacting residues, reflected a high positive correlation motion with a strong negative correlation motion in the loop’s regions. The pocket 2 interacting residues resided between the 550 to 650 amino acids and also showed strong positive correlation motions with the strong negative correlation motion in the loops of pocket 2. These active pocket regions also showed a high positive correlation motion in 1BKA. While apo-Lf (1CB6) showed some positive correlation motion in the pocket 1 region, some loops had regions that showed moderate negative correlation motion. The overall dominated motions in the small molecule attached complexes are positively correlated motions. Thus, the consistent attachment of oxalate ion and CP20 in the active pockets changes the dynamics of Lf.

#### 2.2.9. Gibbs Free Energy Distribution

**Figure 10:**
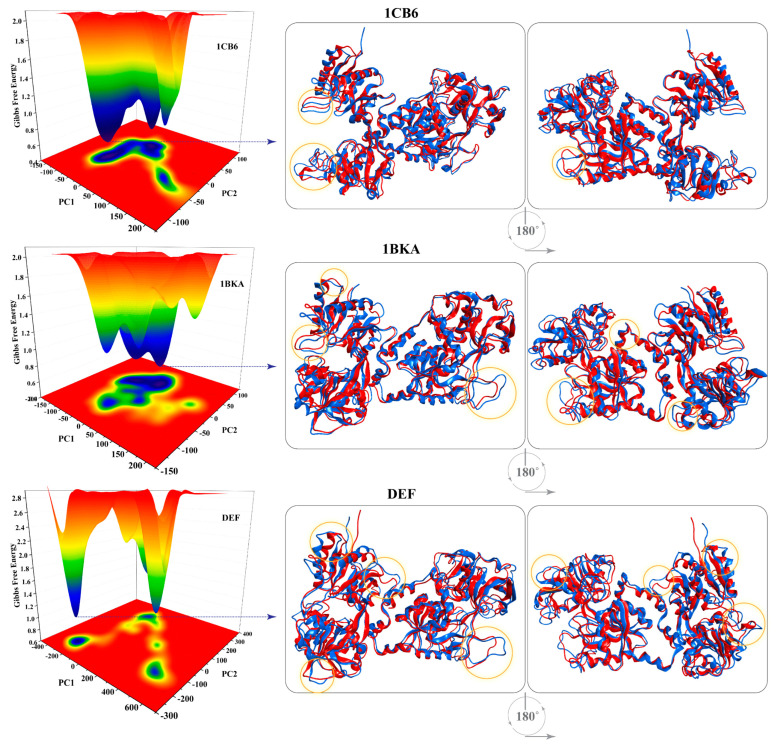
Gibbs free energy plot of 1CB6 (apo-Lf), 1BKA (oxalate-Lf complex), and CP20-Lf complex (DEF). The metastable states are shown in cartoon shape. Blue and red color represents stable energy and high energy state, respectively. The dispersed regions in the superimposed structures are shown in circle.

The free energy landscape (FEL) depicted the transition states of the oxalate ion and CP20 bound complexes and apo-Lf. From each trajectory, the PC1 and PC2 were selected to compute FEL to understand the transition mechanism of the apo state and small inhibitor attached complexes (Figure 10). Low energy conformation from each system was extracted to understand the structural evolution. The apo-Lf showed a significant change in the FEL as compared to the small molecules attached complexes (showed by different colors in the plot). The apo-Lf remained in the low energy level (shown in blue color). The oxalate ion–Lf complex remained mostly in high energy and intermediate energy level, shown in red and yellow color, respectively, and was distributed in many energy states. The CP20 complex remains in high energy levels and show three conformational transitions with high energy levels. The energy of CP20 complex was distributed widely as compared to the apo-Lf which remained in one energy state for most of the simulation time. The high energy levels depict that the attachment of deferiprone with active sites of Lf is strong. During the structure evolution from apo state to small molecule bound states, metastable state’s structure ensembles at different nanosecond times were observed. The metastable (blue) and high energy (red) are presented in cartoon shape in Figure 10. The x-axis and y-axis coordinates with frame number details of the metastable states are tabulated in Supplementary Table S5.

## 3. Materials and Methods

### 3.1. Microscale Thermophoresis (MST) Method

#### 3.1.1. Chemicals

CP20, human Lf 98% lyophilized powder and 2-Amino-2-(hydroxymethyl) propane-1,3-diol (Tris) buffer were purchased from Sigma-Aldrich (Steinheim, Germany). Hydrochloric acid (HCl), and sodium hydroxide (NaOH) were purchased from Merck (Darmstadt, Germany). Ultrapure water was obtained from Arium^®^pro ultrapure water system (Sartorius, Goettingen, Germany).

#### 3.1.2. Instruments

The Monolith NT.115 LabelFree^TM^ MST instrument (NanoTemper Technologies, Munich, Germany) was used to measure the binding events. MST instrument was equipped with a capillary tray allowing for the successive measurement of 16 samples in each run using coated capillaries. A total laser on/off cycle of 35 s, which involves 30 s on and 5 s off was set for each measurement. The MST instrument was supplemented with NT Analysis software provided by NanoTemper Technologies. Fluorescence detection was carried out at excitation wavelength of 280 nm and emission wavelength at 360 nm.

#### 3.1.3. MST Optimization

The measurements were carried out under the optimized MST conditions with LED power 40% (excitation power) and MST power 20% (laser power to induce thermophoresis). MST signals was obtained through 35 s (seconds) of time scale where set laser on for 30 s to induce thermal diffusion followed by 5 s laser off for back diffusion state. Fluorescence was detected at excitation wavelength of 280 nm and emission wavelength at 360 nm.

#### 3.1.4. Samples Preparation

Standard stock solutions were prepared in 0.1 M Tris buffer. Tris buffer was prepared by dissolving 1.2114 g of the powder in water, and then adjusting the pH to 7.4 with HCl and completing the volume to 100 mL with water. The obtained solution was used as a diluent for further stock and samples serial dilutions. CP20 (10 mM) was prepared through dissolving 0.0139 gm of the reference standard powder in 10 mL of 0.1 M tris buffer. While 100 μM of Lf standard solution was prepared by dissolving 0.084 gm of Lf standard powder in 10 mL 0.1 M tris buffer. Then, further dilution was made to reach 1 μM Lf standard solution. The working samples were prepared according to 1:1 serial dilution of CP20 titrated partner in a concentration range of 7 nM to 250 μM then, the fixed concentration of 120 nM of fluorescent partner (Lf) was added to each sample. Mixtures were kept standing for 10 min in dark place to equilibrate before carrying out the MST measurement. 

#### 3.1.5. Data Analysis

The dissociation constant *K_d_* was estimated as follow:

(1) A+B⇌AB

where *A* = binding partner *A*; *B* = binding partner *B*; *AB* = complex

The equilibrium dissociation constant *K_d_* as:

(2) $$K_{d}=\frac{{\left[A\right]}_{free}-{\left[B\right]}_{free}}{\left[AB\right]}$$

Whereas free concentrations of each partner are not known. Total concentrations are used according to the following formula:

(3) $$\left[A\right]={\left[A\right]}_{free}+\left[AB\right]\;and\;\left[B\right]={\left[B\right]}_{free}+\left[AB\right]$$

${\left[A\right]}_{free}$= free concentration of partner A; ${\left[B\right]}_{free}$ = free concentration of partner B; [AB] = bound complex concentration. 

Hence, *K_d_* is calculated as follow:

(4) $$K_{d}=\frac{\left(\left[A\right]-\left[AB\right]\right)\left(\left[B\right]-\left[AB\right]\right)}{\left[AB\right]}$$

Then, fraction bound *FB* was calculated as a total concentration of *A* and *B* and correlated with *K_d_* parameter as follows:

(5) $$FB=\frac{\left[A\right]+\left[B\right]+K_{d}-\sqrt{{\left(\left[A\right]+\left[B\right]+K_{d}\right)}^{2}-4\left[AB\right]}}{2\left[B\right]}$$

where, *FB* represents linearity with normalized fluorescence form MST measurements. 

### 3.2. Computational Analysis

#### 3.2.1. Selection of Crystal Structure of Lf

The crystal structures of Lf attached with the oxalate ion and Fe (+3 charged) (PDB ID: 1BKA, resolution = 2.4 Å) and free state (PDB ID: 1CB6, resolution = 2.0 Å) were retrieved from RCSB Protein Data Bank (https://www.rcsb.org/; accessed on 1 May 2022). The molecular operating environment (MOE, version 2020.0901) structure preparation tool was employed to correct the geometry of the crystal structures using the all atoms Amber14:EHT [24,25] (Amber ff14SB combined with EHT) force field. The topology of the protein, crystallized ligand and Fe ion was refined by adding the missing forcefield parameters, missing atom types, bond stretch parameters, missing angle parameters, and missing van der Waals. The missing residues in the protein structures were modelled using the loop model implemented in MOE. The protein C-N terminals were charged to clarify the start and the end of sequence.

#### 3.2.2. Molecular Docking

To perform the docking of CP20 with the Lf, the ligand structure (CP20) was drawn on ChemDraw as 2D and the 2D structure was then converted to 3D by MOE. Before docking the CP20, the cleaning rules were applied to the ligand structure and the protonation state was optimized using the MMFF94x force field. The geometry of the CP20 was adjusted by adding the missing hydrogens. The MOEs quick preparation tool was employed to calculate the inter-atomic distances, angles, and dihedrals. A 0.01 Kcal/Å2 minimization was performed using the AMBER14:EHT force field to optimize the CP20 structure and calculate the AM1-BCC charges. The Fe ion and the crystallized ligand (oxalate ion) in the Lf define the protein’s two active pockets. The MOE Dock was used to dock the CP20 in both pockets. The oxalate ion in the active site was re-docked and superimposed on the docked conformation to calculate RMSD to validate the docking protocol. The confirmation of the ligand was evaluated and created based on the bond rotation of the atoms to obtain a satisfactory geometry. After having the good geometry of the compound, the triangle matcher method implemented in MOE was used to place the ligand conformations in the active sites of Lf and 100 poses of ligand were scored by London dG scoring function. Finally, maximum 30 poses from 100 were isolated based on the GBVI/WSA dG scoring algorithm. The final complex from the docking was selected based on the docking score, interaction with Lf residues and RMSD of the small molecule with the crystallized ligand. 

#### 3.2.3. Molecular Dynamics Simulation

Three systems were subjected to molecular dynamic simulations including the free state (PDB ID: 1CB6), reference inhibited system (PDB ID: 1BKA), and selected deferiprone docked complex of CP20 with Lf. All the systems were simulated in an implicit solvent model using the AMBER20 [26] GPU implementation Particle Mesh Ewald Molecular Dynamics (PMEMD) [27]. The amino acid-specific forcefield ff19SB implemented in LEap module of AMBER20 was used to generate the topology and coordinate of Lf [26]. The small molecules (ligand) in the complex were parametrized by calculating the AM1-BCC [28] charges with the combination of GAFF2 [28] forcefield. AMBER20s Parmchk2 tool was used to model the missing forcefield parameters. The trivalent and tetravalent cations library [29] for highly charged metal ions was used to treat Fe^3+^ ions in Lf. To neutralize the systems, monovalent ions (Na^+^, Cl^−^) [30] for OPC ions with a concentration of ~0.1 M were added. The LEap module handled missing hydrogens in each system. A truncated octahedral box of optimal point charge (OPC) water model with 10 Å of the buffer distance was added to each system. For long-range electrostatic calculations PMEMD engine on GPUs was used [27]. The Fe^3+^ charge and interaction with the ligand and protein residues was confirmed in the topology and coordinates files of the reference system (PDB ID: 1BKA), and CP20-Lf complex (DEF) using ParmED [31] module of AMBER20. 

The topology and coordinates file for each system was minimized in two steps. The steepest descent minimization of 2000 step was used first, followed by the conjugate gradients minimization for 10,000 steps to remove any bad contacts [32]. All the systems were heated from 0.1 to 300 K in 400 ps time using an NVE ensemble (Microcanonical ensemble) with the combination of Langevin thermostat. For the dynamic propagation, the kinetic energy of harmonic oscillators was adjusted using the Langevin thermostat [33] with 2.0 ps^−1^ frequency of collision. The heating was followed by the 400 ps density adjustment. The systems were equilibrated for 2000 ps at 300 K in NVE ensemble without any restraint selected and pressure relaxation time of 2 ps. The isotropic position scaling algorithm was used to uphold the pressure constant in the equilibration stage with a 1 ps pressure relaxation time. Shake algorithm was applied to constrain all the hydrogen bonds of the systems [34]. The cutoff value of 8 Å was used to compute the long-range electrostatics using the particle-mesh Ewald [35] method. The production run of 110 ns was performed for each system [Apo (PDB ID: 1CB6), reference (PDB ID: 1BKA) and DEF] proceeding the protocols of equilibration. The output trajectory of each system was written after each 10 ps steps for analysis. The output MD trajectories were used to calculate the Cα atom’s Root mean square deviation (RMSD) using the CPPTRAJ module to examine the stability of each simulated system.

**i.**  **Binding Free Energy Calculation**

The Molecular Mechanics/Generalized Born Surface Area (MM/GBSA) was employed to calculate the binding free energy of the protein–ligand complex [36,37,38]. The AMBER20 MMPBSA.py script was used to estimate the binding free energy of the oxalate ion and CP20 bound to Lf. To calculate the binding free energy, 1000 snapshots were selected from the 11,000 frames trajectory with gap of 11 frames. The complex’s binding free energy (Δ*G_bind_*) was calculated using the Equation (1) [39].

$$\Delta G_{bind}=\Delta G_{R+L}\;–\;\left(\Delta G_{R}+\Delta G_{L}\right)\#\left(1\right)$$

The binding free energy of oxalate ion and CP20 complexed with Lf was calculated by Δ*G_R+L_*, while the energy of apo-Lf was calculated by Δ*G_R_* and free energy of oxalate ion and CP20 was estimated by Δ*G_L_*. The complex and apo-states energies of each protein and ligand in equation 1 was calculated by Equation (2).

$$\Delta G=E_{bond}+E_{vdw}+E_{elec}+G_{PB}+G_{SA}\;–\;TS_{S}\#\left(2\right)\;$$

For each system Δ*G*, the bond, angle, and dihedral energies are calculated by *E_bond_*, the van der Waals contributions and electrostatic energies were estimated by the *E_vdw_*, and *E_elec_*. The polar and non-polar interactions were calculated by *G_PB_* and *G_SA_*, while the absolute temperature and solute entropy was calculated by *TS_S_*. The raddi mbondi2 was employed to optimize the topology of the systems and all the energies were calculated in kcal/mol.

**ii.**  **Native Contacts Analysis**

The CPPTRAJ native contacts module was used to detect the relevant amino acid interactions of Lf with the oxalate ion, CP20 and Fe ions with a cutoff distance value of 3.0 Å. Contacts from the first frame were considered as native while after the first frame contacts were selected as change during simulation. The lifetime of each contact was analyzed in selected 11,000 frames. The interactions were analyzed between the residues instead of atoms. The time series analysis of each residue contact was analyzed using the lifetime module of CPPTRAJ.

**iii.**  **Per residue Energy Decomposition**

The total interaction energy of each residue of Lf interacting with the oxalate ion and CP20 was calculated with the per residue energy decomposition (PRED) module of MMPBSA.py script. From each trajectory, 1000 frames were isolated from the 11,000 frames to perform PRED analysis to determine the contribution of each residue to the overall binding energy profile between the Lf-oxalate ion and CP20 complexes. In the total contribution of each residue, the average of internal van der walls and electrostatic energy was selected from the 1000 frames. The side chain and backbone atoms of each residue was decomposed further in the total contribution energy and all the energies were calculated in kcal/mol.

**iv.**  **Residual Fluctuation and Compactness of Lf**

The atomic positional fluctuation of each residue in the Lf was analyzed by means of (RMSF) via CPPTRAJ module of AMBER20 [40]. The flexibility of each residue and change occurred with the attachment of the inhibitor with the protein was analyzed. During simulation, the atomic motion from a shared center of gravity in the Lf structure was computed by radius of gyration (Rg). The overall effect on the compactness of the protein folding caused by the attachment of inhibitor and free state of Lf was observed in Rg.

**v.**  **Dominant Motions in the Lf Structure**

The functional motions in the protein structure due to ligand binding was calculated by principal component analysis (PCA) implemented in AMBER20 module CPPTRAJ [40,41]. In order to observe the domain motions, first the covariance matrix was constructed using the X, Y and Z components of each atom. For each system covariance matrix, 3D positional coordinates ten motion modes were calculated. The internal motions, global rotation and translation of each system was calculated from the covariance matrix. Over the 11,000 frames from the covariance matrix, the eigenvalue and eigenvector were estimated for all atoms except hydrogens and was diagonalized to find the principal components (PCs). The eigenvectors and eigenvalues of the PCs shows the direction and magnitude of motion, respectively. The fraction of each eigenvector was reported in percentage. The motions occurred in the protein was observed by plotting the first two principal components, PC1 and PC2.

**vi.**  **Protein Correlation Motion**

The correlated motion of all residues in each system was analyzed by the dynamic cross-correlation map (DCCM). For each system, a trajectory of 11,000 frames was used to construct the correlation matrix based on the residues of the protein by AMBER20 CPPTRAJ module. The observed motion of a residue in the same direction was reported as positive correlation motion while the residues motions in opposite direction were observed as negative correlation motion [40].

**vii.**  **Gibbs Free Energy Distribution**

For each protein–ligand complex, thermodynamic and protein folding events were observed by the free energy landscape (FEL) using the CPPTRAJ module of AMBER20 [40]. The transitional state and metastable state of the protein during the simulation were observed from the Gibbs free energy values. The Gibbs free energy of the first two principal components (PC1 and PC2) was used and distributed in 100 bins. The highest variation was observed in the PC1 and PC2 components of each system. To distribute the data in bins, the bins with zero population size were maintained with the 0.5 artificial barriers while calculating the Gibbs free energy. The Gibbs free energy was calculated at 300 K temperature and reported in kcal/mol.

**viii.**  **Data Illustration**

Images are illustrated by MOE 2020.0901 [42], VMD [43], Pymol [44], and Blender [45]. The CPPTRAJ was used to generate the lowest energy structure ensembles for each system. All graphs were generated by Origin pro [46].

## 4. Conclusions

The binding affinity between deferiprone and lactoferrin was measured using microscale thermophoresis, and the strong binding results obtained reveal that deferiprone secretion in breast milk can be predicted. Moreover, in microscale thermophoresis technique proved to be valuable in the binding studies realm. In addition, two-fold computational methods including molecular docking and molecular dynamic simulation were employed to predict the binding of deferiprone with the two binding sites of lactoferrin. We observed that Fe plays important role in the binding of deferiprone in both the pockets. In addition, ARG121 and ASP297 of pocket 1 and ASN640 and ASP395 of pocket 2 provide hydrophilic interactions to stabilize deferiprone in those active sites. Subsequently, the molecular dynamic simulation indicates that deferiprone brings energetically stable conformational changes in the structure of the target protein, lactoferrin, and binds with high energy in both pockets due to strong binding interactions; however, deferiprone molecules interact more strongly in pocket 1 than pocket 2. 

## Figures and Tables

**Table 1:** The binding free energy and individual energy components of Lf in complex with oxalate ion and deferiprone.

Complex Name	∆_VDW_ (Kcal/mol)	∆_EEL_ (Kcal/mol)	∆_EGB_ (Kcal/mol)	∆_SASA_ (Å2)	∆G_Total_ (Kcal/mol)
OX1	31.25	−1077.33	917.92	−1.15	−129.32
OX2	23.32	−996.99	848.02	−1.14	−126.78
DF1	−5.33	−119.85	10.23	−2.41	−117.38
DF2	−9.72	−150.81	51.91	−2.91	−111.54

## Data Availability

All data are available from the corresponding author upon reasonable request.

## Supplementary Materials

The following supporting information can be downloaded at: https://www.mdpi.com/article/10.3390/molecules27144604/s1, Table S1: Interactions of Lf with the oxalate ion and deferiprone; Table S2: Contacts analysis of the oxalate ion with lactoferrin with life span of interaction and average distance of the bond; Table S3: Interaction of deferiprone with lactoference with lifetime of each bond and its average distance; Table S4: Per residue energy decomposition of the oxalate ion and deferiprone interactions with the lactoferrin active pockets; Table S5: Metastable state ensemble of each system with its X-Y coordinates, frame number and time(ns) in the trajectory.

## References

[1] Salaris C., Scarpa M., Elli M., Bertolini A., Guglielmetti S., Pregliasco F., Blandizzi C., Brun P., Castagliuolo I. (2021). Protective effects of lactoferrin against SARS-CoV-2 infection in vitro. Nutrients.

[2] Salman B.I., Ibrahim A.E., El Deeb S., Saraya R.E. (2022). Fabrication of novel quantum dots for the estimation of COVID-19 antiviral drug using green chemistry: Application to real human plasma. RSC Adv..

[3] Sharaf Y.A., El Deeb S., Ibrahim A.E., Al-Harrasi A., Sayed R.A. (2022). Two Green Micellar HPLC and Mathematically Assisted UV Spectroscopic Methods for the Simultaneous Determination of Molnupiravir and Favipiravir as a Novel Combined COVID-19 Antiviral Regimen. Molecules.

[4] Rosa L., Tripepi G., Naldi E., Aimati M., Santangeli S., Venditto F., Caldarelli M., Valenti P. (2021). Ambulatory COVID-19 Patients Treated with Lactoferrin as a Supplementary Antiviral Agent: A Preliminary Study. J. Clin. Med..

[5] Campione E., Lanna C., Cosio T., Rosa L., Conte M.P., Iacovelli F., Romeo A., Falconi M., Del Vecchio C., Franchin E. (2021). Lactoferrin as antiviral treatment in COVID-19 management: Preliminary evidence. Int. J. Environ. Res. Public Health.

[6] Miotto M., Di Rienzo L., Bò L., Boffi A., Ruocco G., Milanetti E. (2021). Molecular mechanisms behind anti SARS-CoV-2 action of lactoferrin. Front. Mol. Biosci..

[7] Guo M., Lu X., Wang Y., Brodelius P.E. (2017). Comparison of the interaction between lactoferrin and isomeric drugs. Spectrochim. Acta Part A Mol. Biomol. Spectrosc..

[8] Talebi R., Ahmadi A., Afraz F., Abdoli R. (2016). Parkinson’s disease and lactoferrin: Analysis of dependent protein networks. Gene Rep..

[9] Nurchi V.M., Crisponi G., Lachowicz J.I., Medici S., Peana M., Zoroddu M.A. (2016). Chemical features of in use and in progress chelators for iron overload. J. Trace Elem. Med. Biol..

[10] Anderson B.F., Baker H.M., Dodson E.J., Norris G.E., Rumball S.V., Waters J.M., Baker E.N. (1987). Structure of human lactoferrin at 3.2-A resolution. Proc. Natl. Acad. Sci. USA.

[11] Anderson B.F., Baker H.M., Norris G.E., Rice D.W., Baker E.N. (1989). Structure of human lactoferrin: Crystallographic structure analysis and refinement at 2 8 Å resolution. J. Mol. Biol..

[12] Bailey S., Evans R.W., Garratt R.C., Gorinsky B., Hasnain S., Horsburgh C., Jhoti H., Lindley P.F., Mydin A. (1988). Molecular structure of serum transferrin at 3.3-. ANG. resolution. Biochemistry.

[13] Kolnagou A., Economides C., Eracleous E., Kontoghiorghes G.J. (2006). Low serum ferritin levels are misleading for detecting cardiac iron overload and increase the risk of cardiomyopathy in thalassemia patients. The importance of cardiac iron overload monitoring using magnetic resonance imaging T2 and T2. Hemoglobin.

[14] Martin-Bastida A., Ward R.J., Newbould R., Piccini P., Sharp D., Kabba C., Patel M.C., Spino M., Connelly J., Tricta F. (2017). Brain iron chelation by deferiprone in a phase 2 randomised double-blinded placebo controlled clinical trial in Parkinson’s disease. Sci. Rep..

[15] Boddaert N., Le Quan Sang K.H., Rötig A., Leroy-Willig A., Gallet S., Brunelle F., Sidi D., Thalabard J.-C., Munnich A., Cabantchik Z.I. (2007). Selective iron chelation in Friedreich ataxia: Biologic and clinical implications. Blood J. Am. Soc. Hematol..

[16] Kontoghiorghe C.N., Kolnagou A., Kontoghiorghes G.J. (2013). Potential clinical applications of chelating drugs in diseases targeting transferrin-bound iron and other metals. Expert Opin. Investig. Drugs.

[17] Sooriyaarachchi M., Gailer J. (2010). Removal of Fe3+ and Zn2+ from plasma metalloproteins by iron chelating therapeutics depicted with SEC-ICP-AES. Dalton Trans..

[18] Chakraborty D., Bhattacharyya M. (2000). Deferiprone (L1) induced conformation change of hemoglobin: A fluorescence and CD spectroscopic study. Mol. Cell. Biochem..

[19] Dorraji M.S., Azar V.P., Rasoulifard M. (2014). Interaction between deferiprone and human serum albumin: Multi-spectroscopic, electrochemical and molecular docking methods. Eur. J. Pharm. Sci..

[20] Asmari M., Ratih R., Alhazmi H.A., El Deeb S. (2018). Thermophoresis for characterizing biomolecular interaction. Methods.

[21] Entzian C., Schubert T. (2016). Studying small molecule–aptamer interactions using MicroScale Thermophoresis (MST). Methods.

[22] Jerabek-Willemsen M., Wienken C.J., Braun D., Baaske P., Duhr S. (2011). Molecular interaction studies using microscale thermophoresis. Assay Drug Dev. Technol..

[23] Jerabek-Willemsen M., André T., Wanner R., Roth H.M., Duhr S., Baaske P., Breitsprecher D. (2014). MicroScale Thermophoresis: Interaction analysis and beyond. J. Mol. Struct..

[24] Gerber P.R., Müller K. (1995). MAB, a generally applicable molecular force field for structure modelling in medicinal chemistry. J. Comput.-Aided Mol. Des..

[25] Maier J.A., Martinez C., Kasavajhala K., Wickstrom L., Hauser K.E., Simmerling C. (2015). ff14SB: Improving the accuracy of protein side chain and backbone parameters from ff99SB. J. Chem. Theory Comput..

[26] Case D.A., Belfon K., Ben-Shalom I., Brozell S.R., Cerutti D., Cheatham T., Cruzeiro V.W.D., Darden T., Duke R.E., Giambasu G. (2020). Amber 2020.

[27] Salomon-Ferrer R., Gotz A.W., Poole D., Le Grand S., Walker R.C. (2013). Routine microsecond molecular dynamics simulations with AMBER on GPUs. 2. Explicit solvent particle mesh Ewald. J. Chem. Theory Comput..

[28] Wang B., Merz K.M. (2006). A fast QM/MM (quantum mechanical/molecular mechanical) approach to calculate nuclear magnetic resonance chemical shifts for macromolecules. J. Chem. Theory Comput..

[29] Li P., Song L.F., Merz K.M. (2015). Parameterization of highly charged metal ions using the 12-6-4 LJ-type nonbonded model in explicit water. J. Phys. Chem. B.

[30] Sengupta A., Li Z., Song L.F., Li P., Merz Jr K.M. (2021). Parameterization of Monovalent Ions for the Opc3, Opc, Tip3p-Fb, and Tip4p-Fb Water Models. J. Chem. Inf. Modeling.

[31] Swails J., Hernandez C., Mobley D.L., Nguyen H., Wang L.-P., Janowski P. ParmEd. https://github.com/ParmEd/ParmEd.

[32] Press W.H., Flannery B.P., Teukolsky S.A., Vetterling W.T., Kramer P.B. (1987). Numerical recipes: The art of scientific computing. Phys. Today.

[33] Sindhikara D.J., Kim S., Voter A.F., Roitberg A.E. (2009). Bad seeds sprout perilous dynamics: Stochastic thermostat induced trajectory synchronization in biomolecules. J. Chem. Theory Comput..

[34] Kräutler V., Van Gunsteren W.F., Hünenberger P.H. (2001). A fast SHAKE algorithm to solve distance constraint equations for small molecules in molecular dynamics simulations. J. Comput. Chem..

[35] Darden T., York D., Pedersen L. (1993). Particle mesh Ewald: An N log (N) method for Ewald sums in large systems. J. Chem. Phys..

[36] Hou T., Wang J., Li Y., Wang W. (2011). Assessing the performance of the MM/PBSA and MM/GBSA methods. 1. The accuracy of binding free energy calculations based on molecular dynamics simulations. J. Chem. Inf. Modeling.

[37] Yang T., Wu J.C., Yan C., Wang Y., Luo R., Gonzales M.B., Dalby K.N., Ren P. (2011). Virtual screening using molecular simulations. Proteins Struct. Funct. Bioinform..

[38] Kollman P.A., Massova I., Reyes C., Kuhn B., Huo S., Chong L., Lee M., Lee T., Duan Y., Wang W. (2000). Calculating structures and free energies of complex molecules: Combining molecular mechanics and continuum models. Acc. Chem. Res..

[39] Onufriev A., Bashford D., Case D.A. (2004). Exploring protein native states and large-scale conformational changes with a modified generalized born model. Proteins Struct. Funct. Bioinform..

[40] Roe D.R., Cheatham T.E. (2013). PTRAJ and CPPTRAJ: Software for processing and analysis of molecular dynamics trajectory data. J. Chem. Theory Comput..

[41] Bahar I., Atilgan A.R., Demirel M.C., Erman B. (1998). Vibrational dynamics of folded proteins: Significance of slow and fast motions in relation to function and stability. Phys. Rev. Lett..

[42] ULC C.C.G. (2020). Molecular Operating Environment (MOE), 2020.09.

[43] Humphrey W., Dalke A., Schulten K. (1996). VMD: Visual molecular dynamics. J. Mol. Graph..

[44] Schrödinger L. (2015). The PyMOL Molecular Graphics System, Version 1.8.

[45] Community B.O. (2018). Blender—A 3D Modelling and Rendering Package.

[46] (2021). OriginPro Software.

