# Supplementary material for: Microscale Thermophoresis and Molecular Modelling to Explore the Chelating Drug Transportation in the Milk to Infant
Source: Molecules. 2022 Jul 19;27(14):4604. doi: 10.3390/molecules27144604 (PMC9319837; doi:10.3390/molecules27144604)
Supplement: Supplementary file 1 [file molecules-27-04604-s001.zip › molecules-1786553-supplementary.pdf]

Supplementary Materials

Microscale Thermophoresis and Molecular Modelling to Explore the Chelating Drug Transportation in the Milk to Infant

Mufarreh Asmari [1,†], Muhammad Waqas [2,†], Adel Ehab Ibrahim [2,3], Sobia Ahsan Halim [2], Ajmal Khan [2], Ahmed Al-Harrasi [2], Hermann Wätzig [4] and Sami El Deeb [2,4,*]

[1] College of Pharmacy, King Khalid University, Abha 62529, Saudi Arabia; masmri@kku.edu.sa
[2] Natural and Medical Sciences Research Center, University of Nizwa, P.O. Box 33, Birkat Al Mauz,
Nizwa 616, Oman; mwaqas@unizwa.edu.om (M.W.); adel.ehab@pharm.psu.edu.eg (A.E.I.); sobia_halim@unizwa.edu.om (S.A.H.); ajmalkhan@unizwa.edu.om (A.K.); aharrasi@unizwa.edu.om (A.A.-H.)
[3] Analytical Chemistry Department, Faculty of Pharmacy, Port-Said University, Port Fouad 42526, Egypt
[4] Institute of Medicinal and Pharmaceutical Chemistry, Technische Universität Braunschweig, 38106 Braunschweig, Germany; h-waetzig@tu-bs.de
* Correspondence: s.eldeeb@tu-bs.de
† These authors contributed equally to this work.

Contents

Supplementary Table S1: Interactions of Lf with the oxalate-ion and deferiprone.
Supplementary Table S2: Contacts analysis of the Oxalate ion with Lactoferrin with life span of interaction and average distance of the bond.
Supplementary Table S3: Interaction of Deferiprone with Lactoference with lifetime of each bond and its average distance.
Supplementary Table S4: Per-residue energy decomposition of the oxalate-ion and defereprone interactions with the lactoferin active pockets.
Supplementary Table S5: Metastable state ensemble of each system with its X-Y coordinates, frame number and time(ns) in the trajectory.

Table S1: Interactions of Lf with the oxalate-ion and deferiprone

Pocket	Ligand Atoms	Receptor Atoms	Residues	Interaction	Distance (Å)	E (kcal/mol)
OX1	O3	OG1	THR117	H-acceptor	2.70	-1.9
	O3	N	GLY124	H-acceptor	2.81	-1.9
	O4	NE	ARG121	H-acceptor	2.75	-1.7
	O4	NH2	ARG121	H-acceptor	2.89	-3.5
	FE	OD2	ASP 60	Metal	1.93	-3.8
OX2	O1	N	ALA 467	H-acceptor	2.92	-0.5
	O3	OG1	THR461	H-acceptor	2.60	-1.7
	O3	N	GLY468	H-acceptor	2.89	-3.2
	O4	NE	ARG465	H-acceptor	2.86	-2.3
	O1	N	ALA467	H-acceptor	2.92	-0.5
	O1	N	ALA467	H-acceptor	2.92	-0.5
	FE	OD2	ASP395	Metal	1.96	-4.1
DF1	O8	O	ARG121	H-donor	2.61	-1.2
	C10	OD2	ASP297	H-donor	3.05	-0.7
	FE	OG1	THR122	Metal	2.06	-2.4
	FE	OD2	ASP60	Ionic	1.93	-17.6
	FE	OD1	ASP60	Ionic	1.96	-16.9
DF2	C10	O	ASN640	H-donor	3.11	-0.5
	O7	OD2	ASP395	Ionic	2.1	-7.1
	FE	OD2	ASP395	Metal	1.90	-2.5
	FE	NE2	HIS597	Metal	2.08	-4.3
	FE	OD2	ASP395	Ionic	1.90	-18.2

Table S2: Contacts analysis of the Oxalate ion with Lactoferrin with life span of interaction and average distance of the bond

S. No.	Residue Atom: Ligand Atom	No of frames	Fraction	Average Distance (Å)
1	:690@FE_:OX1@O1	11000	100.00%	1.92
2	:691@FE_:OX2@O1	11000	100.00%	1.92
3	:690@FE_:OX1@O2	11000	100.00%	1.92
4	:691@FE_:OX2@O2	11000	100.00%	1.93
5	:690@FE_:OX1@C2	11000	100.00%	2.75
6	:690@FE_:OX1@C1	11000	100.00%	2.75
7	:691@FE_:OX2@C1	11000	100.00%	2.77
8	:691@FE_:OX2@C2	11000	100.00%	2.78
9	:190@OD1_:OX1@O1	10987	99.90%	2.68
10	:119@NE_:OX1@O4	6842	62.20%	2.82
11	:119@NH2_:OX1@O4	5892	53.60%	2.84
12	:464@OD1_:OX2@O1	4118	37.40%	2.8
13	:463@NE_:OX2@O4	3318	30.20%	2.81
14	:526@OH_:OX2@O1	1455	13.20%	2.83
15	:458@OG1_:OX2@O3	1375	12.50%	2.72
16	:462@OD1_:OX2@O2	1239	11.30%	2.8
17	:595@NE2_:OX2@O2	1226	11.10%	2.86
18	:90@OD2_:OX1@O2	1063	9.66%	2.71
19	:463@NH2_:OX2@O4	976	8.87%	2.86
20	:433@OH_:OX2@O1	896	8.15%	2.9
21	:525@OH_:OX2@O2	663	6.03%	2.89
22	:525@CZ_:OX2@O2	587	5.34%	2.89
23	:90@OH_:OX1@O1	222	2.02%	2.92
24	:121@N_:OX1@O3	6	0.05%	2.87
25	:115@OG1_:OX1@O3	3	0.03%	2.6
26	:190@CE2_:OX1@O2	3	0.03%	2.8
27	:190@CZ_:OX1@O2	3	0.03%	2.83
28	:190@OH_:OX1@O1	3	0.03%	2.84
29	:190@OH_:OX1@O2	3	0.03%	2.91
30	:190@CZ_:OX1@C2	1	0.01%	2.99

Table S3: Interaction of Deferiprone with Lactoference with lifetime of each bond and its average distance

S.No	Residue Atom: Ligand Atom	No of frames	Fraction	Average Distance (Å)
1	:690@FE_:DF1@O8	11000	100.00%	1.96
2	:691@FE_: DF2@O8	11000	100.00%	1.98
3	:81@OD1_: DF1@O8	10662	96.90%	2.72
4	:642@ND1_: DF2@O8	5998	54.50%	2.88
5	:645@OD2_: DF2@O8	3888	35.30%	2.66
6	:528@OH_: DF2@O8	758	6.89%	2.9
7	:81@OE2_: DF1@O7	722	6.56%	2.61
8	:93@OH_: DF1@O8	505	4.59%	2.81
9	:83@OH_: DF1@O7	96	0.87%	2.92
10	:464@OG1_: DF2@O7	2	0.02%	2.91
11	:643@O_:DF2@C10	2	0.02%	2.93

Table S4: Per-residue energy decomposition of the oxalate-ion and deferiprone interactions with the lactoferin active pockets

Complex	Residues	Van der walls	Electrostatics	Polar Solvation	ΔG Total
OX1	TYR 90	0.287	-8.279	4.279	-3.713
	THR 115	-0.139	-3.402	-3.402	-6.943
	ARG 119	0.376	-81.049	67.049	-13.624
	THR 120	0.184	-8.397	-2.397	-10.61
	ALA 121	-0.096	-3.836	-1.133	-5.065
	TYR 190	1.762	-13.758	3.758	-8.238
	FE 690	16.037	-511.231	437.137	-58.057
OX2	LEU 433	-0.007	-4.201	-0.915	-5.123
	ALA 459	-0.074	-1.816	1.701	-0.189
	THR 463	-0.783	-6.018	3.728	-3.073
	THR 526	-0.01	-7.542	2.473	-5.079
	ALA 595	-0.007	0.889	-0.625	0.257
	FE 691	14.191	-408.593	350.346	-44.056
DF1	ASP 61	-0.204	12.866	-18.557	-5.895
	GLU 81	1.06	1.753	-8.4	-5.587
	VAL 82	-0.01	0.019	-0.044	-0.035
	TYR 83	-0.091	-2.149	-0.123	-2.363
	TYR 93	-0.443	-3.061	1.081	-2.423
	HIE 254	-0.693	2.611	-3.415	-1.497
	FE 690	5.534	-147.997	111.889	-30.574
DF2	GLY 398	-0.016	-0.166	0.15	-0.032
	ALA 464	1.012	-3.096	2.144	0.06
	THR 531	-0.952	-7.351	-1.015	-9.318
	LEU 643	-0.466	-1.323	-0.631	-2.42
	FE 691	4.901	-157.784	124.389	-28.494
All the energies are calculated in kcal/mol					

Table S5: Metastable state ensemble of each system with its X-Y coordinates, frame number and time (ns) in the trajectory

System	X-coordinates	Y-Coordinates	Frame No	Time (ns)
1CB6	-40.431	-34.217	2833	28.33
1BKA	44.569	-38.058	5737	57.37
DEF	-271.765	-198.410	1009	10.09
